# Supplementary material for: SK4 K+ channels are therapeutic targets for the treatment of cardiac arrhythmias
Source: EMBO Mol Med. 2017 Feb 20;9(4):415–29. doi: 10.15252/emmm.201606937 (PMC5376763; doi:10.15252/emmm.201606937)
Supplement: Supplementary file 1 — Appendix [file EMMM-9-415-s001.pdf]

Appendix

<u>***Table of contents***</u>**:**

1.Mathematical model (p.1)

1.1. Appendix: Mathematical Modelling (p.1)

1.2. References (p.16)

2. Supplementary Figure Legends (p.18)

3. Appendix Supplementary Figures (p.21)

1.Mathematical model

Using mathematical modelling, the SK4 K^+ conductance contribution to SAN firing rate was examined. For that purpose the mathematical model of mouse SAN designed by Kharche et al. (Kharche et al., 2011) was implemented and the SK4 current (I_{SK4}) was added to the modelling. The contribution of I_{SK4} was analyzed by simulating the effect of the SK4 channel blocker TRAM-34 and compared to the experimental findings. For the mathematical modelling of I_{SK4} we used from the Ca^{2+}-dependent sensitivity curve of SK4 channel activation, $n_x = 2.7$ (the slope of the Hill curve) and k_x=0.27 μM (Ca^{2+} dissociation constant for SK4 activation) as measured by Logsdon et al. (Logsdon et al., 1997). In addition, the opening of SK channels has been shown to be the result of a sequence of events starting with Ca^{2+} binding to calmodulin followed by conformational changes of calmodulin and SK channels that will result in structural rearrangements of the pore-occluding gate domain (Berkefeld et al., 2010). The time constant of the activation process (τ_a) was assumed to 5 ms under Ca^{2+} saturation. Channel deactivation, initiated upon dissociation of Ca^{2+}, is reflected by the deactivation time constant (τ_d), which is assumed to range between 15 and 60 ms (Hirschberg et al., 1998; Pedarzani et al., 2001; Xia et al., 1998). We used $\tau_a = 5$ ms and $\tau_d = 50$ ms for the activation and deactivation time constants, respectively. Finally, we assumed that the maximal amplitude reached by I_{SK4} was 1.8 pA/pF, which corresponds to the current measured in the present paper at +20 mV.

<u>1.1. Appendix: Mathematical Modelling</u>

a. Introduction

We extended the computational model of mouse SANCs function from Kharche et al. (Kharche et al., 2011) to include the SK4 ion channel denoted I_{SK4}. The intracellular concentration of K^+ and Na^+ were assumed to be constant and the SK4 channel formulation was added.

b. Mice model

It is assumed that the net current passing through the membrane is determined by the following equation:

$$(1)\quad I = I_{Na,1.1} + I_{Na,1.5} + I_{CaL,1.2} + I_{CaL,1.3} + I_{CaT} + I_{Kr} + I_{Ks} + I_f + I_{to} + I_{sus} + I_{K1} + I_{NaK} + I_{NaCa} + I_{b,Na} + I_{b,K} + I_{b,Ca} + I_{SK4},$$

with, $I_{Na,1.1}$ the Na^+ channel isoform Nav1.1 current, $I_{Na,1.5}$ the Na^+ channel isoform Nav1.5 current, $I_{CaL,1.2}$ the L-type Ca^{2+} channel isoform Cav1.2 current, $I_{CaL,1.3}$ the L-type Ca^{2+} channel isoform Cav1.3 current, I_{CaT} the T-type Ca^{2+} current, I_{Kr} the rapid delayed rectifying K^+ current, I_{Ks} the slow delayed rectifying K^+ current, I_f the hyperpolarization-activated current, I_{to} the transient component of 4-AP-sensitive currents, I_{sus} the sustained component of 4-AP-sensitive currents, I_{K1} the rapid delayed rectifying K^+ current, I_{NaK} the Na^+-K^+ pump current, I_{NaCa} the Na^+/Ca^{2+} exchanger current, $I_{b,Na}$ the background Na^+ current, $I_{b,K}$ background K^+ current, $I_{b,Ca}$ the background Ca^{2+} current and I_{SK4} the calcium-activated potassium channel SK4.

c. Equations

Formulation of I_{SK4}

SK channels typically show little or no voltage dependence (Latorre et al., 1989). However, an increase in Ca^{2+} concentration ($[Ca^{2+}_{sub}]$) leads to an increase in the open probability of the channel (f_{SK}). It is assumed that the relationship between the Ca^{2+} concentration and the steady-state open probability follows a Hill equation (McManus, 1991; Silva et al., 2007). The Hill equation can empirically be described by two parameters: the slope of the curve (n_x) and the midpoint of the curve (k_x). Steady-state Ca^{2+} dependent value of f_{SK} gate:

$$f_{SK,\propto} = \frac{[Ca_{sub}^{2+}]^{nx}}{[Ca_{sub}^{2+}]^{nx} + kx^{nx}}$$

Time constant – assumed to be only dependent on whether the channels are operating during diastolic depolarization or repolarization.

During diastolic depolarization:

$$\tau_f = 5\,ms$$

During repolarization:

$$\tau_f = 50\,ms$$

Current formulation:

$$I_{SK4} = C \cdot g_{SK4} \cdot f_{SK} \cdot (V_m - E_K)$$

$$\frac{df_{SK}}{dt} = \frac{(f_{SK,\infty} - f_{SK})}{\tau_f}$$

Model constants

**Model Parameter**	**Value**	**Definition**
$\boldsymbol{C}$, **pF**	25	Cell electric capacitance
**F, C/M**	96485	The Faraday constant
**T, K**	310.5	The absolute temperature for 37˚C
**R, J/(M K)**	8.314472	Universal gas constant
$\mathbf{V_{cell}}$, **pL**	3	Cell volume
$\mathbf{V_{sub}}$, **pL**	0.03328117	Subspace volume
$\mathbf{V_{jSR}}$, **pL**	0.0036	Volume of the JSR
$\mathbf{V_{nSR}}$, **pL**	0.0348	Volume of NSR (Ca^{2+} uptake store)
$\mathbf{V}_{\boldsymbol{i}}$, **pL**	1.34671883	Myoplasmic volume
$[\boldsymbol{Mg}^{2+}]_{\boldsymbol{i}}$, **mM**	2.5	Intracellular Mg^{2+}
$[\boldsymbol{Na}^{+}]_{\boldsymbol{o}}$, **mM**	140	Extracellular Na^{+}

$[Ca^{2+}]_o$, mM	1.8	Extracellular Ca^{2+}
$[K^+]_o$, mM	5.4	Extracellular K^+
$[Na^+]_i, mM$	8.1179761505	Intracellular Na^+ concentration
$[K^+]_i, mM$	139.8854603066	Intracellular K^+ concentration
$g_{st}, nS/pF$	0.0024	Normalized conductance for I_{st} channels
E_{st}, mV	17	Apparent reversal potential of I_{st}
$g_{bNa}, nS/pF$	0.00486	Normalized conductance for I_{bNa} channels
$g_{K1}, nS/pF$	0.0324	Normalized conductance for I_{K1} channels
$g_{Ks}, nS/pF$	0.01196	Normalized conductance for I_{Ks} channels
E_{CaL}, mV	47	Reversal potential of I_{CaL}
K_{mfCa}, mM	0.00035	Dissociation constant of Ca^{2+}-dependent inactivation of I_{CaL}
α_{fCa}, ms^{-1}	0.021	Ca^{2+} dissociation rate constant for I_{CaL}
E_{CaT}, mV	45	Reversal potential of I_{CaT}
$g_{sus}, nS/pF$	0.0156	Normalized conductance for I_{sus} channels
$I_{NaKmax}, pA/pF$	5.698	Maximal Na/K pump current conductance
K_{mNa}, mM	14	Half-maximal $[Na^+]_i$for I_{NaK}
K_{mK}, mM	1.4	Half-maximal $[K^+]_o$ for I_{NaK}
K_{1ni}, mM	395.3	Dissociation constant for $[Na^+]_i$ binding to the first site on I_{NCX} transporter
K_{1no}, mM	1628	Dissociation constant for $[Na^+]_o$ binding to first site on I_{NCX} transporter
K_{2ni}, mM	2.289	Dissociation constant for $[Na^+]_i$ binding to second site on I_{NCX} transporter
K_{2no}, mM	561.4	Dissociation constant for $[Na^+]_o$ binding to second site on I_{NCX} transporter
K_{3ni}, mM	26.44	Dissociation constant for $[Na^+]_i$ binding to third site on I_{NCX} transporter
K_{3no}, mM	4.663	Dissociation constant for $[Na^+]_o$ binding to third site on I_{NCX} transporter

$\mathbf{K_{ci}, mM}$	0.0207	Dissociation constant for $[Ca^{2+}]_i$ binding on I_{NCX} transporter
$\mathbf{K_{co}, mM}$	3.663	Dissociation constant for $[Ca^{2+}]_o$ binding to I_{NCX} transporter
$\mathbf{K_{cni}, mM}$	26.44	Dissociation constant for $[Na^{+}]_i$ and $[Ca^{2+}]_i$ simultaneous binding to I_{NCX} transporter
$\mathbf{Q_{ci}}$	0.1369	Fractional charge movement during the $[Ca^{2+}]_{sub}$ occlusion reaction of the I_{NCX} transporter
$\mathbf{Q_{co}}$	0	Fractional charge movement during the $[Ca^{2+}]_o$ occlusion reaction of the I_{NCX}transporter
$\mathbf{Q_{n}}$	0.4315	Fractional charge movement during Na^+ occlusion reactions of the I_{NCX}transporter
$\mathbf{\tau_{difCa}, ms}$	0.04	Time constant of Ca^{2+} diffusion from the submembrane to myoplasm
$\mathbf{TC_{tot}, mM}$	0.031	Total concentration of the troponin-Ca^{2+} site
$\mathbf{TMC_{tot}, mM}$	0.062	Total concentration of the troponin-Mg^{2+} site
$\mathbf{k_{fTC}, mM^{-1} \cdot ms^{-1}}$	88.8	Ca^{2+} association constant of troponin
$\mathbf{k_{fTMC}, mM^{-1} \cdot ms^{-1}}$	237.7	Ca^{2+} association constant of the troponin-Mg site
$\mathbf{k_{bTC}, ms^{-1}}$	0.446	Ca^{2+} dissociation constant for the troponin-Ca site
$\mathbf{k_{bTMC}, ms^{-1}}$	0.00751	Ca^{2+} dissociation constant for the troponin-Mg site
$\mathbf{k_{fTMM}, mM^{-1} \cdot ms^{-1}}$	2.277	Mg^{2+} association constant for the troponin-Mg site
$\mathbf{k_{bTMM}, ms^{-1}}$	0.751	Mg^{2+} dissociation constant for the troponin-Mg site

$\mathbf{CM_{tot}}, \mathbf{mM}$	0.045	Total calmodium concentration
$\mathbf{k_{fCM}}, \mathbf{mM^{-1} \cdot ms^{-1}}$	237.7	Ca^{2+} association constant for calsequestrin
$\mathbf{k_{bCM}}, \mathbf{ms^{-1}}$	0.542	Ca^{2+} dissociation constant for calmodulin
$\mathbf{CQ_{tot}}, \mathbf{mM}$	10	Total calsequestrin concentration
$\boldsymbol{k}_{\mathbf{fCQ}}, \mathbf{mM^{-1} \cdot ms^{-1}}$	0.534	Ca^{2+} association constant for calsequestrin
$\mathbf{k_{bCQ}}, \mathbf{1/ms}$	0.445	Dissociation constant for calsequestrin
$\mathbf{k_{oCa}}, \mathbf{1/(mM^2 \cdot ms})$	10	Baseline non-SR-dependent transition rate constant for the RyR
$\mathbf{k_{om}}, \mathbf{ms^{-1}}$	0.06	Rate transition constant for RyR
$\mathbf{k_{iCa}}, \mathbf{mM^{-1} \cdot ms^{-1}}$	0.5	RyR Ca^{2+} dependent inactivation rate
$\mathbf{k_{im}}, \mathbf{ms^{-1}}$	0.005	Rate transition constant for RyR
$\mathbf{EC_{50SR}}, \mathbf{mM}$	0.45	EC50 for Ca^{2+} SR-dependent activation of SR Ca^{2+} release
**MaxSR**	15	Ca^{2+} modeling parameter
**MinSR**	1	Ca^{2+} modeling parameter
**HSR**	2.5	Parameter for Ca^{2+}-dependent activation of SR Ca^{2+} release
$\mathbf{n_{up}}$	2	SR Ca^{2+} uptake and Hill coefficient
$\mathbf{g_{Na15}}, \boldsymbol{nS/pF}$	0.000237	Normalized conductance for I_{Na15} channels
$\mathbf{g_{Na11}}, \boldsymbol{nS/pF}$	0.000237	Normalized conductance for I_{Na11} channels
$\mathbf{g_{CaL12}}, \boldsymbol{nS/pF}$	0.24	Normalized conductance for I_{Na12} channels
$\mathbf{g_{CaL13}}, \boldsymbol{nS/pF}$	0.72	Normalized conductance for I_{Na13} channels
$\mathbf{g_{CaT}}, \boldsymbol{nS/pF}$	0.5586	Normalized conductance for I_{CaT} channels
$\mathbf{g_{If}}, \boldsymbol{nS/pF}$	0.228	Normalized conductance for I_{If} channels
$\mathbf{g_{Kr}}, \boldsymbol{nS/pF}$	0.09456	Normalized conductance for I_{Kr} channels

$\mathbf{g_{to}}, \boldsymbol{nS/pF}$	0.1968	Normalized conductance for I_{to} channels
$\mathbf{g_{SK4}}, \boldsymbol{nS/pF}$	0.0195	Normalized conductance for I_{SK4} channels
$\mathbf{K_{NaCa}}, \boldsymbol{pA/pF}$	220	Scaling factor for I_{NaCa}
$\mathbf{P_{up}}, \mathbf{mM/ms}$	0.04	Rate constant for Ca^{2+} uptake by the Ca^{2+} pump in the network SR
$\mathbf{k}_{\boldsymbol{s}}, \boldsymbol{ms^{-1}}$	130e4	Release rate parameter
$\mathbf{K_{mf}}, \boldsymbol{mM}$	0.00008	Forward-mode Ca^{2+} affinity of the SERCA pump
$\mathbf{K_{mr}}, \boldsymbol{mM}$	4.5	Reverse-mode Ca^{2+} affinity of the SERCA pump
$\mathbf{g_{bCa}}, \boldsymbol{nS/pF}$	0.0006	Normalized conductance for I_{bCa} channels
$\mathbf{g_{bK}}, \boldsymbol{nS/pF}$	0.001	Normalized conductance for I_{bK} channels
$\boldsymbol{\tau}_{\mathbf{tr}}, \mathbf{ms}$	40	Time constant for Ca^{2+} transport from the network to junctional SR
$\mathbf{E}_{\boldsymbol{T}}, \mathbf{mV}$	1000·(R·T/F)	
$\mathbf{K}_{\boldsymbol{up}}, \mathbf{mM}$	0.0006	Half-maximal $[Ca^{2+}]_i$ for Ca^{2+} uptake in the NSR
$\boldsymbol{E_{Na15}}, \boldsymbol{mV}$	41.5761	Reversal potential of $I_{Na1.5}$

Initial conditions

Model Parameter	Value	Definition
$\mathbf{V}_{\boldsymbol{m}}, \mathbf{mV}$	-64.5216286940	Membrane potential
$\boldsymbol{q_a}$	0.6246780312	Activation gating variable of I_{st}
$\boldsymbol{q_i}$	0.4537033169	Inactivation gating variable of I_{st}
$\boldsymbol{d_T}$	0.0016256324	Inactivation gate of I_{CaT}
$\boldsymbol{f_T}$	0.4264459666	Activation gate of I_{CaT}
$\boldsymbol{p_a}$	0.4043600437	Activation gating variable of I_{Kr}
$\boldsymbol{p_i}$	0.9250035423	Inactivation gating variable of I_{Kr}

$\boldsymbol{x_s}$	0.0127086259	Activation gating variable of I_{Ks}
$\boldsymbol{f_{L,1.2}}$	0.9968141226	Inactivation gate for $I_{CaL,1.2}$
$\boldsymbol{d_{L,1.2}}$	0.0000045583	Activation gate for $I_{CaL,1.2}$
$\boldsymbol{f_{L,1.3}}$	0.9809298233	Inactivation gate for $I_{CaL,1.3}$
$\boldsymbol{d_{L,1.3}}$	0.0002036671	Activation gate for $I_{CaL,1.3}$
$\boldsymbol{f_{Ca}}$	0.7649576191	Ca^{2+}-dependent inactivation gating variable for $I_{CaL,1.2}$ and $I_{CaL,1.2}$
$\boldsymbol{r}$	0.0046263658	Activation gating variable of I_{to} and I_{sus}
$\boldsymbol{q}$	0.6107148187	Inactivation gating variable of I_{to}
$\boldsymbol{m_{1.5}}$	0.4014088304	Activation gating variable of Nav1.5
$\boldsymbol{h_{1.5}}$	0.2724817537	Fast inactivation gating variable of Nav1.5
$\boldsymbol{j_{1.5}}$	0.0249208708	Slow inactivation gating variable of Nav1.5
$\boldsymbol{m_{1.1}}$	0.1079085266	Activation gating variable of Nav1.1
$\boldsymbol{h_{1.1}}$	0.4500098710	Fast inactivation gating variable of Nav1.1
$\boldsymbol{j_{1.1}}$	0.0268486392	Slow inactivation gating variable of Nav1.1
$\boldsymbol{y}$	0.0279984462	Activation gating variable of I_f
$[\boldsymbol{Ca^{2+}}]_i, \boldsymbol{mM}$	0.0000319121	Intracellular Ca^{2+} concentration or Ca^{2+} concentration in the cytosol
$[\boldsymbol{Ca^{2+}}]_{jSR}, \boldsymbol{mM}$	0.1187281829	Ca^{2+} concentration in the JSR
$[\boldsymbol{Ca^{2+}}]_{nSR}, \boldsymbol{mM}$	1.5768287365	Ca^{2+} concentration in the NSR
$[\boldsymbol{Ca^{2+}}]_{sub}, \boldsymbol{mM}$	0.0000560497	Ca^{2+} concentration in the subspace
$\boldsymbol{f_{TC}}$	0.0063427103	Fractional occupancy of the troponin Ca^{2+} site by $[Ca^{2+}]_i$
$\boldsymbol{f_{TMC}}$	0.1296677919	Fractional occupancy of the troponin Mg^{2+} site by $[Ca^{2+}]_i$
$\boldsymbol{f_{TMM}}$	0.7688656371	Fractional occupancy of the troponin Mg^{2+} site by Mg^{2+}
$\boldsymbol{f_{CMs}}$	0.0242054739	Fractional occupancy of calmodulin by $[Ca^{2+}]_{sub}$

$\boldsymbol{f_{CMi}}$	0.0138533048	Fractional occupancy of calmodulin by $[Ca^{2+}]_i$
$\boldsymbol{f_{CQ}}$	0.1203184861	Fractional occupancy of calsequestrin by $[Ca^{2+}]_{rel}$
$\boldsymbol{R}$	0.7720290515	Fraction of reactivated (closed) RyR channels
$\boldsymbol{OO}$	0.0000000760	Open fraction of RyR channels
$\boldsymbol{S}$	0.0000000213	Inactive fraction of RyR channels
$\boldsymbol{RI}$	0.2162168926	Fraction of RyR inactivated channels

Gating variables

$$dg/dt = (g_\infty - g)/\tau_g$$

For any gating variable g with steady state g_∞

4-aminopyridine-sensitive currents, $\boldsymbol{I_{to}}$and $\boldsymbol{I_{sus}}$

$$I_{to} = C \cdot g_{to} \cdot (V_m - E_K) \cdot q \cdot r$$

$$I_{sus} = C \cdot g_{sus} \cdot (V_m - E_K) \cdot r$$

$$q_\infty = 1/(1 + \exp((V_m + 49.0)/13.0))$$
$$r_\infty = 1/(1 + \exp(-(V_m - 19.3)/15.0))$$
$$\tau_q = (6.06 + 39.102/(0.57 \cdot \exp(-0.08 \cdot (V_m + 44.0)) + 0.065 \cdot \exp(0.1 \cdot (V_m + 45.93))))/0.67$$
$$\tau_r = (2.75 + 14.40516/(1.037 \cdot \exp(0.09 \cdot (V_m + 30.61)) + 0.369 \cdot \exp(-0.12 \cdot (V_m + 23.84))))/0.303$$

Ca^{2+} background current, $\boldsymbol{I_{bCa}}$

$$I_{bCa} = C \cdot g_{bCa} \cdot (V_m - E_{Ca})$$

K^+ background current, $\boldsymbol{I_{bK}}$

$$I_{bK} = C \cdot g_{bK} \cdot (V_m - E_K)$$

Na^+ background current, $\boldsymbol{I_{bNa}}$

$$I_{bNa} = C \cdot g_{bNa} \cdot (V_m - E_{Na})$$

L-type channel current, $\boldsymbol{I_{CaL}}$

$$I_{CaL12} = C \cdot g_{CaL12} \cdot (V_m - E_{CaL}) \cdot d_{L12} \cdot f_{L12} \cdot f_{Ca}$$
$$I_{CaL13} = C \cdot g_{CaL13} \cdot (V_m - E_{CaL}) \cdot d_{L13} \cdot f_{L13} \cdot f_{Ca}$$

$$I_{CaL} = I_{CaL12} + I_{CaL13}$$

$$d_{\infty 12} = 1/(1 + \exp(-(V_m + 3.0)/5.0))$$
$$f_{\infty 12} = 1/(1 + \exp((V_m + 36.0)/4.6))$$
$$d_{\infty 13} = 1/(1 + \exp(-(V_m + 13.5)/6))$$
$$f_{\infty 13} = 1/(1 + \exp((V_m + 35.0)/7.3))$$
$$f_{Ca\infty} = K_{mfCa}/(K_{mfCa} + \mathrm{Ca}_{sub})$$
$$\alpha_{dL} = -28.39 \cdot (V_m + 35)/(\exp(-(V_m + 35)/2.5) - 1) - 84.9 \cdot V_m./(\exp(-0.208 \cdot V_m) - 1)$$
$$\beta_{dL} = 11.43 \cdot (V_m - 5.0)./(\exp(0.4 \cdot (V_m - 5.0)) - 1)$$
$$\tau_{dL} = 2000/(\alpha_{dL} + \beta_{dL})$$
$$\tau_{fl} = (7.4 + 45.77 \cdot \exp(-0.5 \cdot (V_m + 28.1) \cdot (V_m + 28.1)/(11 \cdot 11)))$$
$$\tau_{fCa} = f_{Ca\infty}/\alpha_{fCa}$$

T-type Ca^{2+} current, $\boldsymbol{I_{CaT}}$

$$I_{CaT} = C \cdot g_{CaT} \cdot (V_m - E_{CaT}) \cdot d_T \cdot f_T$$

$$d_{T\infty} = 1/(1 + \exp(-(V_m + 26)/6))$$
$$f_{T\infty} = 1/(1 + \exp((V_m + 61.7)/5.6))$$
$$\tau_{dT} = 1/(1.068 \cdot \exp((V_m + 26.3)/30) + 1.068 \cdot \exp(-(V_m + 26.3)/30))$$
$$\tau_{fT} = 1/(0.0153 \cdot \exp(-(V_m + 61.7)/83.3) + 0.015 \cdot \exp((V_m + 61.7)/15.38))$$

Hyperpolarization-activated, funny current, I_f

$$I_{fNa} = C \cdot 0.3833 \cdot \mathrm{g}_{If} \cdot (V_m - E_{Na}) \cdot \mathrm{y}$$
$$I_{fK} = C \cdot 0.6167 \cdot \mathrm{g}_{If} \cdot (V_m - E_K) \cdot \mathrm{y}$$

$$y_\infty = 1/(1 + \exp((V_m + 106.8)/16.3))$$
$$\tau_y = 1.5049/(\exp(-(V_m + 590.3) \cdot 0.01094) + \exp((V_m - 85.1)/17.2))$$

K1 current, I_{K1}

$$I_{K1} = C \cdot g_{K1} \cdot 1/(1 + \exp(0.070727 \cdot (V_m - E_K))) \cdot ([K^+]_o/([K^+]_o + 0.228880)) \cdot (V_m - E_K)$$

Kr current, I_{Kr}

$$I_{Kr} = C \cdot g_{Kr} \cdot (V_m - E_K) \cdot p_a \cdot p_i$$

$$p_{a\infty} = 1/(1 + \exp(-(V_m + 21.173694)/9.757086))$$
$$p_{i\infty} = 1/(1 + \exp((V_m + 20.758474 - 4.0)/(19.0)))$$

$$\tau_{pa} = 0.699821/(0.003596 \cdot \exp((V_m)/15.339290) + 0.000177 \cdot \exp(-(V_m)/25.868423))$$
$$\tau_{pi} = 0.2 + 0.9 \cdot 1.0/(0.1 \cdot \exp(V_m/54.645) + 0.656 \cdot \exp(V_m/106.157))$$

Ks current, I_{Ks}

$$I_{Ks} = C \cdot g_{Ks} \cdot (V_m - E_{Ks}) \cdot xs^2$$

$$xs_{\infty} = 1/(1 + \exp(-(V_m - 20.876040)/11.852723))$$
$$\tau_{xs} = 1000/(13.097938/(1 + \exp(-(V_m - 48.910584)/10.630272)) + \exp(-V_m/35.316539))$$

Sodium current, I_{Na}

$$FNa = (9.52\mathrm{e}-02 \cdot \exp(-6.3\mathrm{e}-2 \cdot (V_m + 34.4))/(1 + 1.66 \cdot \exp(-0.225 \cdot (V_m + 63.7)))) + 8.69\mathrm{e}-2$$
$$hs_{11} = (1 - \mathrm{FNa}) \cdot \mathrm{h11} + \mathrm{FNa} \cdot \mathrm{j11}$$
$$\mathrm{hs}_{15} = (1 - \mathrm{FNa}) \cdot \mathrm{h15} + \mathrm{FNa} \cdot \mathrm{j15}$$

$$I_{Na11} = C \cdot g_{Na11} \cdot m_{11}^3 \cdot hs_{11} \cdot V_m \cdot [Na]_0 \cdot F/(E_T \cdot 1000) \cdot (\exp((V_m - \mathrm{E}_{Na})/E_T) - 1)/(\exp(V_m/E_T) - 1)$$
$$I_{Na15} = C \cdot g_{Na15} \cdot m_{15}^3 \cdot hs_{15} \cdot V_m \cdot [Na]_0 \cdot F/(E_T \cdot 1000) \cdot (\exp((V_m - E_{\mathrm{Na15}})/E_T) - 1)/(\exp(V_m/E_T) - 1)$$

$$I_{Na} = I_{Na11} + I_{Na15}$$

$$m_{11,\infty} = 1/(1 + \exp(-(V_m + 36.097331 - 5.0)/5.0))\text{^}(1/3)$$
$$h_{11,\infty} = 1/(1 + \exp((V_m + 56.0)/3.0))$$
$$j_{11,\infty} = h_{11,\infty}$$

$$m_{15,\infty} = 1/(1 + \exp(-(V_m + 45.213705)/7.219547))\text{^}(1/3)$$
$$h_{15,\infty} = 1/(1 + \exp(-(V_m + 62.578120)/(-6.084036)))$$
$$j_{15,\infty} = h_{15,\infty}$$

$$\tau_{m11} = 1000 \cdot ((0.6247\mathrm{e}-03/(0.832 \cdot \exp(-0.335 \cdot (V_m + 56.7)) + 0.627 \cdot \exp(0.082 \cdot (V_m + 65.01)))) + 0.0000492)$$

$$\tau_{h11} = 1000.0 \cdot (((3.717e-06 \cdot \exp(-0.2815 \cdot (V_m + 17.11)))/(1 + 0.003732 \cdot \exp(-0.3426 \cdot (V_m + 37.76)))) + 0.0005977)$$

$$\tau_{j11} = 1000 \cdot (((0.00000003186 \cdot \exp(-0.6219 \cdot (V_m + 18.8)))/(1 + 0.00007189 \cdot \exp(-0.6683 \cdot (V_m + 34.07)))) + 0.003556)$$

$$\tau_{m15} = \tau_{m11}$$
$$\tau_{h15} = \tau_{h11}$$
$$\tau_{j15} = \tau_{j11}$$

Na^+ - K^+ pump current, I_{NaK}

$$I_{NaK} = C \cdot I_{NaKmax} \cdot ((([K^+]_o).\hat{}1.2)./((\mathrm{K}_{mK}).\hat{}1.2 + ([K^+]_o).\hat{}1.2)) \cdot (([Na^+]_i.\hat{}1.3)./((\mathrm{K}_{\mathrm{mNa}}).\hat{}1.3 + ([Na^+]_i).\hat{}1.3))./(1.0 + \exp(-(V_m - \mathrm{E}_{Na} + 120)/30))$$

Na^+-Ca^{2+} exchanger current, I_{NCX}

$$d_0 = 1 + ([Ca^{2+}]_o/\mathrm{K}_{co}) \cdot (1 + \exp(\mathrm{Q}_{co} \cdot V_m/\mathrm{E}_T)) + ([Na^+]_o/K_{1no}) \cdot (1 + ([Na^+]_o/\mathrm{K}_{\mathrm{2no}}) \cdot (1 + [Na^+]_o/\mathrm{K}_{\mathrm{3no}}))$$

$$k_{43} = [Na^+]_i/(\mathrm{K}_{\mathrm{3ni}} + [Na^+]_i)$$
$$\mathrm{k}_{41} = \exp(-\mathrm{Q}_n \cdot V_m/(2 \cdot \mathrm{E}_T))$$
$$\mathrm{k}_{34} = [Na^+]_o/(\mathrm{K}_{\mathrm{3no}} + [Na^+]_o)$$
$$\mathrm{k}_{21} = ([Ca^{2+}]_o/\mathrm{K}_{\mathrm{co}}) \cdot \exp(Q_{co} \cdot V_m/\mathrm{E}_T)/\mathrm{do};$$
$$\mathrm{k}_{23} = ([Na^+]_o/\mathrm{K}_{\mathrm{1no}}) \cdot ([Na^+]_o/\mathrm{K}_{\mathrm{2no}}) \cdot (1 + [Na^+]_o./\mathrm{K}_{\mathrm{3no}}) \cdot \exp(-\mathrm{Q}_n \cdot V_m./(2 \cdot \mathrm{E}_T))./d_0$$
$$\mathrm{k}_{32} = \exp(\mathrm{Q}_n \cdot V_m./(2 \cdot \mathrm{E}_T))$$
$$x_1 = \mathrm{k}_{34} \cdot \mathrm{k}_{41} \cdot (\mathrm{k}_{23} + \mathrm{k}_{21}) + \mathrm{k}_{21} \cdot \mathrm{k}_{32} \cdot (\mathrm{k}_{43} + \mathrm{k}_{41})$$

$$d_i = 1 + ([Ca^{2+}]_{sub}/\mathrm{K}_{ci}) \cdot (1 + \exp(-\mathrm{Q}_{\mathrm{ci}} \cdot V_m/\mathrm{E}_T) + [Na^+]_i/\mathrm{K}_{\mathrm{cni}}) + ([Na^+]_i/\mathrm{K}_{\mathrm{1ni}}) \cdot (1 + ([Na^+]_i/\mathrm{K}_{\mathrm{2ni}}) \cdot (1 + [Na^+]_i/\mathrm{K}_{\mathrm{3ni}}))$$

$$k_{12} = ([Ca^{2+}]_{sub}/\mathrm{K_{ci}}) \cdot \exp(-\mathrm{Q_{ci}} \cdot V_m/\mathrm{E}_T)/\mathrm{d}_i$$

$$k_{14} = ([Na^{+}]_{i}/\mathrm{K_{1ni}}) \cdot ([Na^{+}]_{i}/\mathrm{K_{2ni}}) \cdot (1 + [Na^{+}]_{i}/\mathrm{K_{3ni}}) \cdot \exp(\mathrm{Q}_n \cdot V_m./(2 \cdot \mathrm{E}_T))./\mathrm{d}_i$$

$$x_2 = \mathrm{k_{43}} \cdot \mathrm{k_{32}} \cdot (\mathrm{k_{14}} + \mathrm{k_{12}}) + \mathrm{k_{41}} \cdot \mathrm{k_{12}} \cdot (\mathrm{k_{34}} + \mathrm{k_{32}})$$

$$x_3 = \mathrm{k_{43}} \cdot \mathrm{k_{14}} \cdot (\mathrm{k_{23}} + \mathrm{k_{21}}) + \mathrm{k_{12}} \cdot \mathrm{k_{23}} \cdot (\mathrm{k_{43}} + \mathrm{k_{41}})$$

$$x_4 = \mathrm{k_{34}} \cdot \mathrm{k_{23}} \cdot (\mathrm{k_{14}} + \mathrm{k_{12}}) + \mathrm{k_{21}} \cdot \mathrm{k_{14}} \cdot (\mathrm{k_{34}} + \mathrm{k_{32}})$$

$$I_{NCX} = C \cdot K_{NaCa} \cdot (k_{21} \cdot x_2 - k_{12} \cdot x_1)/(x_1 + x_2 + x_3 + x_4)$$

Sustained inward current, I_{st}

$$I_{st} = C \cdot g_{st} \cdot (V_m - E_{st}) \cdot q_a \cdot q_i$$

$$q_{a,\infty} = 1/(1 + \exp(-(V_m + 67)/5))$$

$$\alpha_{qa} = 1/(0.15 \cdot \exp(-V_m/11) + 0.2 \cdot \exp(-V_m/700))$$

$$\beta_{qa} = 1/(16 \cdot \exp(V_m/8) + 15 \cdot \exp(V_m/50))$$

$$\tau_{qa} = 1/(\alpha_{qa} + \beta_{qa})$$

$$\alpha_{qi} = 0.15 \cdot 1/(3100 \cdot \exp((V_m + 10)/13) + 700.3 \cdot \exp((V_m + 10)/70))$$

$$\beta_{qi} = 0.15 \cdot 1/(95.7 \cdot \exp(-(V_m + 10)/10) + 50 \cdot \exp(-(V_m + 10)/700)) + 0.000229/(1 + \exp(-(V_m + 10)/5))$$

$$q_{i,\infty} = \alpha_{qi}/(\alpha_{qi} + \beta_{qi})$$

$$\tau_{qi} = 1/(\alpha_{qi} + \beta_{qi})$$

Reversal potential

$$E_{Na} = E_T \cdot \log([Na^{+}]_o/[Na^{+}]_i)$$

$$E_K = E_T \cdot \log([K^{+}]_o/[K^{+}]_i)$$

$$E_{Ks} = E_T \cdot \log(([K^{+}]_o + 0.12 \cdot [Na^{+}]_o)/([K^{+}]_i + 0.12 \cdot [Na^{+}]_i))$$

$$E_{Ca} = (E_T/2) \cdot \log([Ca^{2+}]_o/[Ca^{2+}]_{sub})$$

Ca^{2+} fluxes in the SR

$$j_{SRCarel} = k_s \cdot OO \cdot ([Ca^{2+}]_{jSR} - [Ca^{2+}]_{sub})$$

$$k_{CaSR} = MaxSR - (MaxSR - MinSR)/(1 + (EC_{50SR}/[Ca^{2+}]_{jSR})^{HSR})$$

$$k_{oSRCa} = k_{oCa}/k_{CaSR}$$

$$k_{iSRCa} = k_{iCa} \cdot k_{CaSR}$$

$$dR/dt = (k_{im} \cdot RI - k_{iSRCa} \cdot [Ca^{2+}]_{sub} \cdot R) - (k_{oSRCa} \cdot [Ca^{2+}]_{sub}^2 \cdot R - k_{om} \cdot OO)$$

$$dOO/dt = (k_{oSRCa} \cdot [Ca^{2+}]_{sub}^2 \cdot R - k_{om} \cdot OO) - (k_{iSRCa} \cdot [Ca^{2+}]_{sub} \cdot OO - k_{im} \cdot S)$$

$$dS/dt = (k_{iSRCa} \cdot [Ca^{2+}]_{sub} \cdot OO - k_{im} \cdot S) - (k_{om} \cdot S - k_{oSRCa} \cdot [Ca^{2+}]_{sub}^2 \cdot RI)$$

$$dRI/dt = (k_{om} \cdot S - k_{oSRCa} \cdot [Ca^{2+}]_{sub}^2 \cdot RI) - (k_{im} \cdot RI - k_{iSRCa} \cdot [Ca^{2+}]_{sub} \cdot R)$$

Intracellular Ca^{2+} flux

$$kkkk = ([Ca^{2+}]_i/K_{mf})^{n_{up}} - ([Ca^{2+}]_{nSR}/K_{mr})^{n_{up}}$$

$$j_{up} = P_{up} \cdot (kkkk/(1 + kkkk))$$

$$j_{Cadiff} = ([Ca^{2+}]_{sub} - [Ca^{2+}]_i)/\tau_{difCa}$$

$$j_{tr} = ([Ca^{2+}]_{nSR} - [Ca^{2+}]_{jSR})/\tau_{diftr}$$

Ca^{2+} buffering

$$df_{TC} = k_{fTC} \cdot [Ca^{2+}]_i \cdot (1 - f_{TC}) - k_{bTC} \cdot f_{TC}$$

$$df_{TMC} = k_{fTMC} \cdot [Ca^{2+}]_i \cdot (1 - f_{TMC} - f_{TMM}) - k_{bTMC} \cdot f_{TMC}$$

$$df_{TMM} = k_{fTMM} \cdot [Mg^{2+}]_i \cdot (1 - f_{TMC} - f_{TMM}) - k_{bTMM} \cdot f_{TMM}$$

$$df_{CMi} = k_{fCM} \cdot [Ca^{2+}]_i \cdot (1 - f_{CMi}) - k_{bCM} \cdot f_{CMi}$$

$$df_{CMs} = k_{fCM} \cdot [Ca^{2+}]_{sub} \cdot (1 - f_{CMs}) - k_{bCM} \cdot f_{CMs}$$

$$df_{CQ} = k_{fCQ} \cdot [Ca^{2+}]_{jSR} \cdot \left(1 - f_{CQ}\right) - k_{bCQ} \cdot f_{CQ}$$

Dynamics of Ca^{2+} concentrations in cell compartments

$$d[Ca^{2+}]_i/dt = ((j_{Cadif} \cdot V_{sub} - j_{up} \cdot V_{nSR})/V_i) - (CM_{tot} \cdot (df_{CMi}/dt) + TC_{tot} \cdot (df_{TC}/dt) + TCM_{tot} \cdot (df_{TMC}/dt))$$

$$d[Ca^{2+}]_{sub}/dt = (-(I_{CaL} + I_{CaT} + I_{bCa} - 2 \cdot I_{NCX})/(2 \cdot \mathrm{F}) + j_{SRCarel} \cdot V_{jSR})/V_{sub} - j_{Cadiff} - \mathrm{CM}_{tot} \cdot \mathrm{df}_{\mathrm{CMs}}$$

$$d[Ca^{2+}]_{jSR}/dt = j_{tr} - j_{SRCarel} - CQ_{tot} \cdot df_{CQ}$$

$$d[Ca^{2+}]_{nSR}/dt = j_{up} - j_{tr} \cdot V_{jSR}/V_{nSR}$$

1.2. References

Berkefeld, H., Fakler, B., and Schulte, U. (2010). Ca2+-activated K+ channels: from protein complexes to function. Physiol Rev 90: 1437-1459.

Hirschberg, B., Maylie, J., Adelman, J.P., and Marrion, N.V. (1998). Gating of recombinant small-conductance Ca-activated K+ channels by calcium. J Gen Physiol 111:565-581.

Kharche, S., Yu, J., Lei, M., and Zhang, H. (2011). A mathematical model of action potentials of mouse sinoatrial node cells with molecular bases. Am J Physiol Heart Circ Physiol 301: H945-963.

Latorre, R., Oberhauser, A., Labarca, P., and Alvarez, O. (1989). Varieties of calcium-activated potassium channels. Annu Rev Physiol 51: 385-399.

Logsdon, N.J., Kang, J., Togo, J.A., Christian, E.P., and Aiyar, J. (1997). A novel gene, hKCa4, encodes the calcium-activated potassium channel in human T lymphocytes. J Biol Chem 272: 32723-32726.

McManus, O.B. (1991). Calcium-activated potassium channels: regulation by calcium. J Bioenerg Biomembr 23: 537-560.

Pedarzani, P., Mosbacher, J., Rivard, A., Cingolani, L.A., Oliver, D., Stocker, M., Adelman, J.P., and Fakler, B. (2001). Control of electrical activity in central neurons by modulating the gating of small conductance Ca2+-activated K+ channels. J Biol Chem 276: 9762-9769.

Silva, H.S., Kapela, A., and Tsoukias, N.M. (2007). A mathematical model of plasma membrane electrophysiology and calcium dynamics in vascular endothelial cells. Am J Physiol Cell Physiol 293: C277-293.

Xia, X.M., Fakler, B., Rivard, A., Wayman, G., Johnson-Pais, T., Keen, J.E., Ishii, T., Hirschberg, B., Bond, C.T., Lutsenko, S., *et al.* (1998). Mechanism of calcium gating in small-conductance calcium-activated potassium channels. Nature 395: 503-507.

2. Supplementary figure Legends

Appendix Figure S1: TRAM-34 does not interfere with voltage-gated Ca^{2+} currents, I_f and NCX currents in hESC-CMs.

A. Representative trace of a voltage-ramp protocol before and after applying 5 μM TRAM-34. Cells were held at -20 mV and a voltage ramp of 150 ms from −90 to +60 mV was applied. The experiments were performed in zero free Ca^{2+} in the pipet solution (n = 7).

B. Sensitivity of the I_f current to 5 μM TRAM-34. Shown are current-density voltage relations (n = 3-9). Cells were held at -40 mV and the membrane voltage was stepped from -40 mV to -100 mV.

C. Representative traces showing in the same cell the sensitivity of the I_{NCX} current before and after exposure to 5 µM TRAM-34, followed by washout and exposure to 3 µM KB-R7943. A voltage ramp protocol was performed from +50 mV to -100 mV for 215 ms. The effects of 3 µM KB-R7943 and 5 µM TRAM-34 were examined using the voltage ramp protocol and quantified as percentage of inhibition from control at -100 mV and +50 mV (n = 3-9).

Appendix Figure S2: SK4 K^+ channels modulate the firing rate in WT mouse SAN cells.

A and B. Representative traces of spontaneous APs recorded in SAN cells under the indicated conditions.

C. Data summary of TRAM-34 (2 µM) on MDP and firing rate. Two-Tailed paired *t*-test. TRAM-34 significantly decreased the spontaneous firing rate (*P =0.0446, n = 5) and depolarized the MDP (**P =0.0013, n = 5).

Appendix Figure S3: Blockade of SK4 channels by clotrimazole improves the ECG arrhythmic features of CASQ2-D307H KI and CASQ2 KO mice under rest conditions

A. Representative ECG recording following intraperitoneal injection of vehicle (upper) and 20 mg/kg clotrimazole (lower) in WT mice at rest. Sequential vehicle and clotrimazole injections were performed on the same animal.

B. Data summary of heart rate (Paired *t*-test; *P =0.0364, n = 10) and PR interval (Paired *t*-test; *P =0.0437, n = 10) in WT mice at rest.

C. Representative ECG recording following IP injection of vehicle (upper) and 20 mg/kg clotrimazole (lower) in CASQ2-D307H KI mice at rest.

D. Data summary of heart rate (Paired *t*-test; *P =0.0260, n = 7) and PR interval (P not significant, n = 7) in CASQ2-D307H KI mice at rest.

E. Representative ECG recording following IP injection of vehicle (upper) and 20 mg/kg clotrimazole (lower) in CASQ2 KO mice at rest.

F. Data summary of heart rate (Paired *t*-test; **P =0.0078, n = 7) and PR interval (Paired *t*-test; *P = 0.0111, n = 7) in CASQ2 KO mice at rest.

Appendix Figure S4: Blockade of SK4 channels by clotrimazole improves the ECG arrhythmic features of CASQ2-D307H KI and CASQ2 KO mice during treadmill exercise.

A. Representative ECG recording following intraperitoneal injection of vehicle (upper) and 20 mg/kg clotrimazole (lower) in WT mice during treadmill exercise.

B. Data summary of heart rate (Paired *t*-test; *P = 0.0229, n = 10) and PR interval (P not significant, n = 10).

C. Representative ECG recording following IP injection of vehicle (upper) and 20 mg/kg clotrimazole (lower) in CASQ2-D307H KI mice during treadmill exercise. Note how clotrimazole changed the non-sustained ventricular tachycardia (NSVT) into ventricular premature complex (VPC).

D. Data summary of heart rate (Paired t-test; **P =0.004, n = 7) and PR interval (Paired t-test; *P = 0.0305, n = 7) in CASQ2-D307H KI mice during treadmill exercise.

E. Representative ECG recording following IP injection of vehicle (upper) and 20 mg/kg clotrimazole (lower) in CASQ2 KO mice during exercise.

F. Data summary of heart rate (Paired t-test; **P =0.0037, n = 7) and PR interval (Paired t-test; *P = 0.0394, n = 7) in CASQ2 KO mice during exercise.

Appendix Figure S5: Model predictions with or without I_{SK4} gating time constants.

A-D. Model predictions with (blue traces) and without (orange traces) the contribution of I_{SK4}. When I_{SK4} was added to the model, the channel activation and deactivation time constants were constrained to τ_a = 5 ms and τ_d = 50 ms, respectively. From the Ca^{2+}-dependent sensitivity curve of SK4 channel activation measured by Logsdon et al. [2], we constrained the model with a Hill slope of $n_x = 2.7$ and a Ca^{2+} dissociation constant of $k_x = 0.27$ µM.

E-H. Model predictions with (blue traces) and without (orange traces) the contribution of I_{SK4}. The model was constrained with a Hill slope of $n_x = 2.7$ and a Ca^{2+} dissociation constant of $k_x =$ 0.27 µM but the gating time constants were removed from the equations. Action potential: Vm (A,E), heart rate: HR (B, F), SK4 current density: I_{SK4} (C, G) and Ca^{2+} concentration in the submembrane space: $[Ca^{2+}]_{sub}$. Note the important drop in the HR when removing the contribution of I_{SK4}.

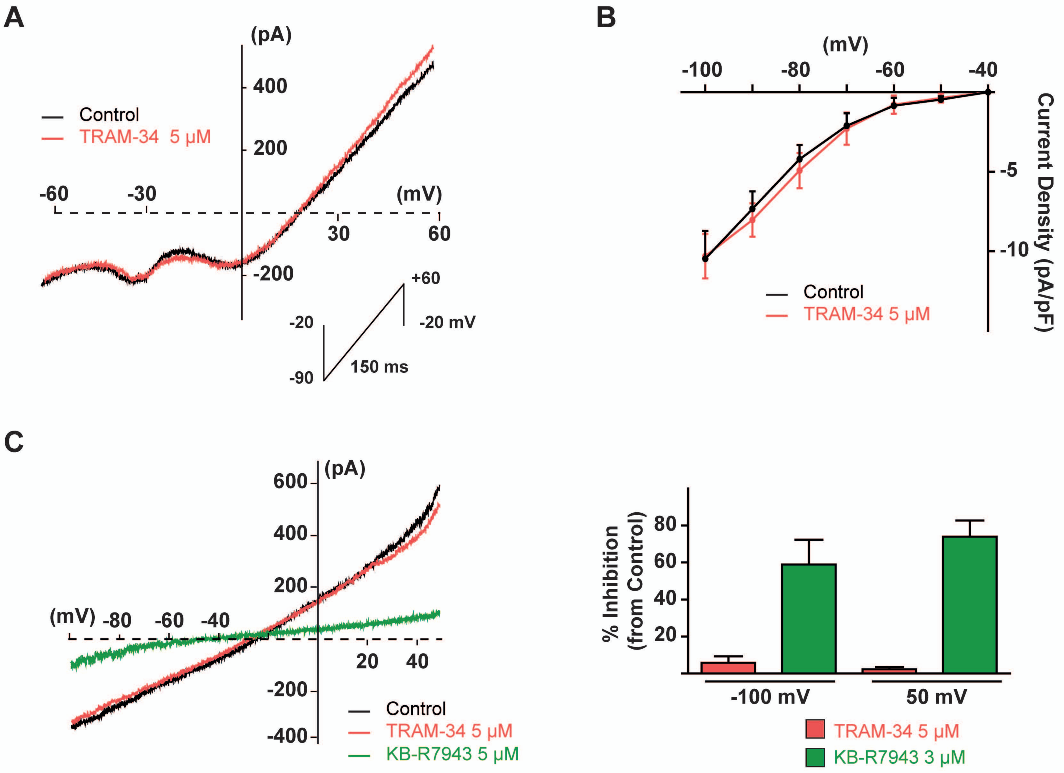
A
(pA)
400
200
-200
Control
TRAM-34 5 μM
-60
-30
30
60
(mV)
+60
-20
-20 mV
-90
150 ms
B
(mV)
-100
-80
-60
-40
-5
-10
Current Density (pA/pF)
Control
TRAM-34 5 μM
C
(pA)
600
400
200
-200
-400
(mV)
-80
-60
-40
20
40
Control
TRAM-34 5 μM
KB-R7943 5 μM
% Inhibition (from Control)
80
60
40
20
-100 mV
50 mV
TRAM-34 5 μM
KB-R7943 3 μM

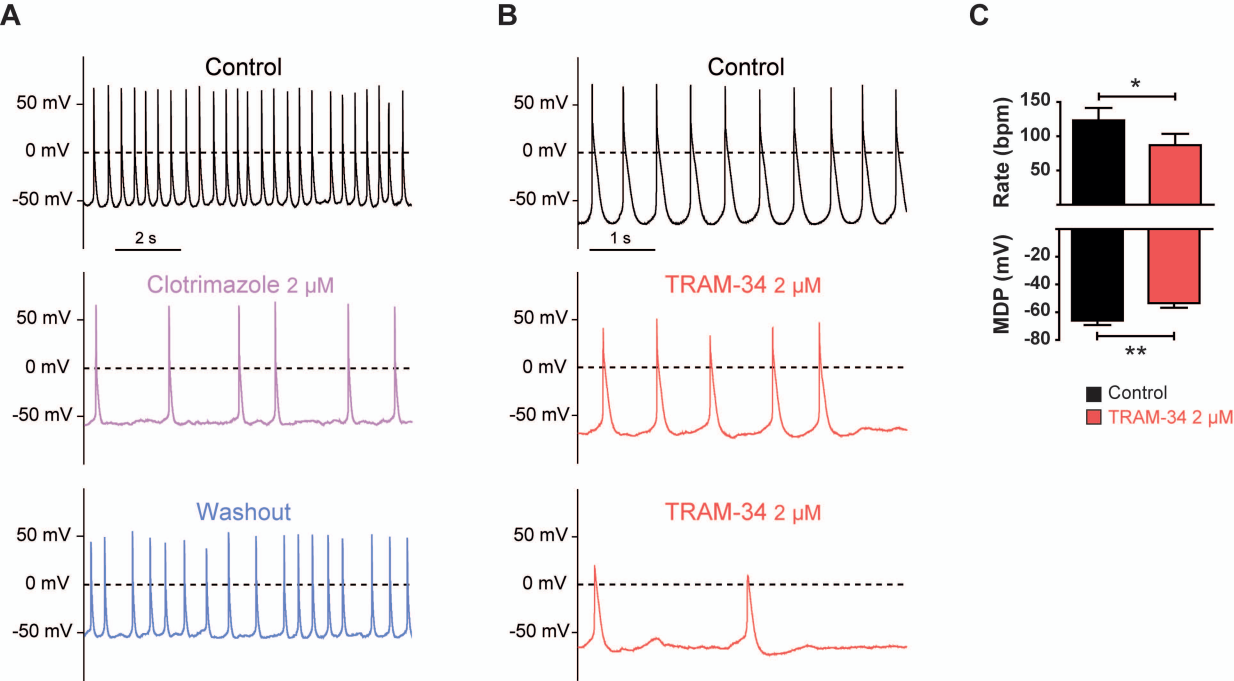
A
Control
50 mV
0 mV
-50 mV
2 s
Clotrimazole 2 µM
50 mV
0 mV
-50 mV
Washout
50 mV
0 mV
-50 mV
B
Control
50 mV
0 mV
-50 mV
1 s
TRAM-34 2 µM
50 mV
0 mV
-50 mV
TRAM-34 2 µM
50 mV
0 mV
-50 mV
C
Rate (bpm)
150
100
50
*
MDP (mV)
-20
-40
-60
-80
**
Control
TRAM-34 2 µM

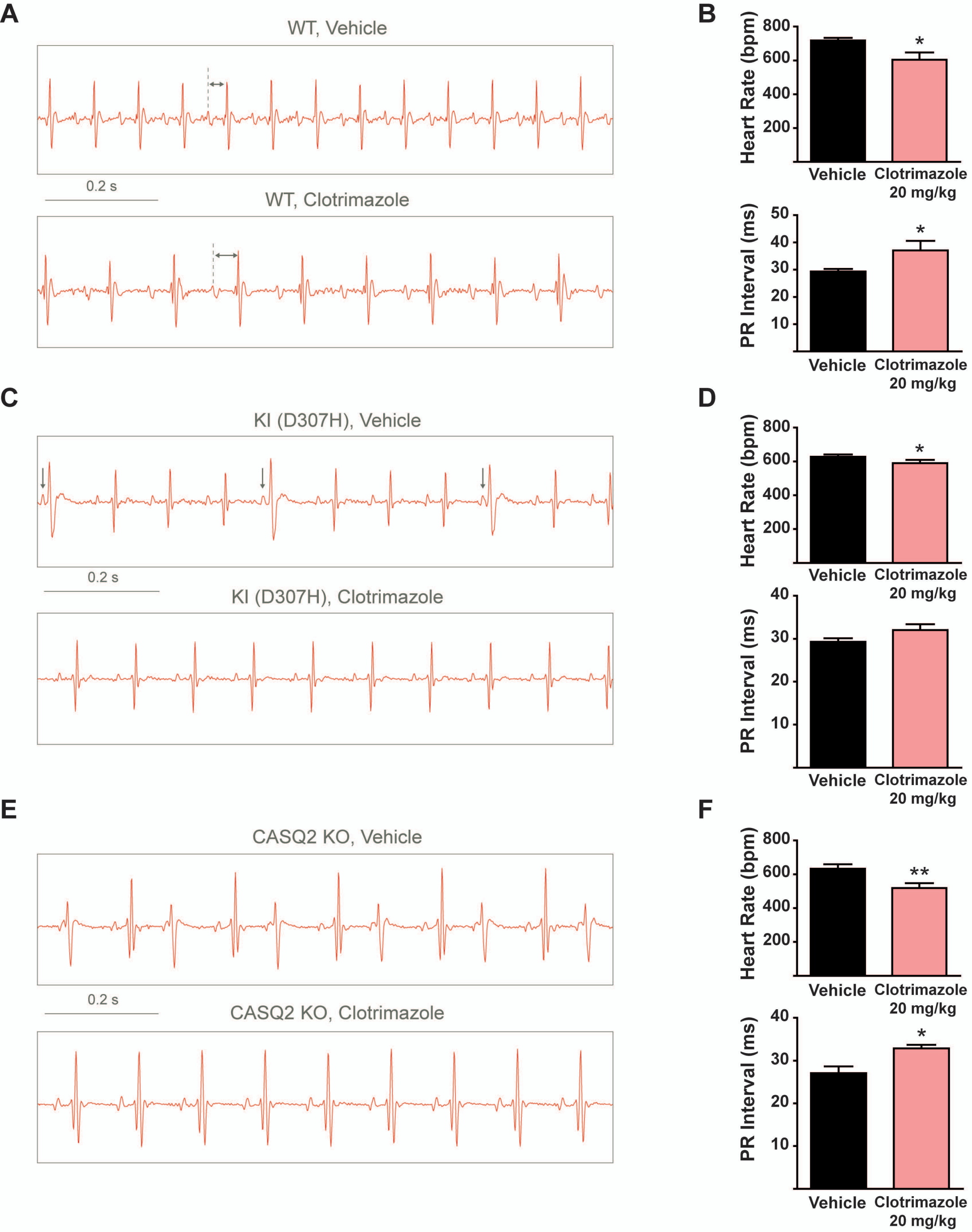

A
WT, Vehicle
0.2 s
WT, Clotrimazole
B
Heart Rate (bpm)
800
600
400
200
*
Vehicle
Clotrimazole 20 mg/kg
PR Interval (ms)
50
40
30
20
10
*
Vehicle
Clotrimazole 20 mg/kg
C
KI (D307H), Vehicle
0.2 s
KI (D307H), Clotrimazole
D
Heart Rate (bpm)
800
600
400
200
*
Vehicle
Clotrimazole 20 mg/kg
PR Interval (ms)
40
30
20
10
Vehicle
Clotrimazole 20 mg/kg
E
CASQ2 KO, Vehicle
0.2 s
CASQ2 KO, Clotrimazole
F
Heart Rate (bpm)
800
600
400
200
**
Vehicle
Clotrimazole 20 mg/kg
PR Interval (ms)
40
30
20
10
*
Vehicle
Clotrimazole 20 mg/kg

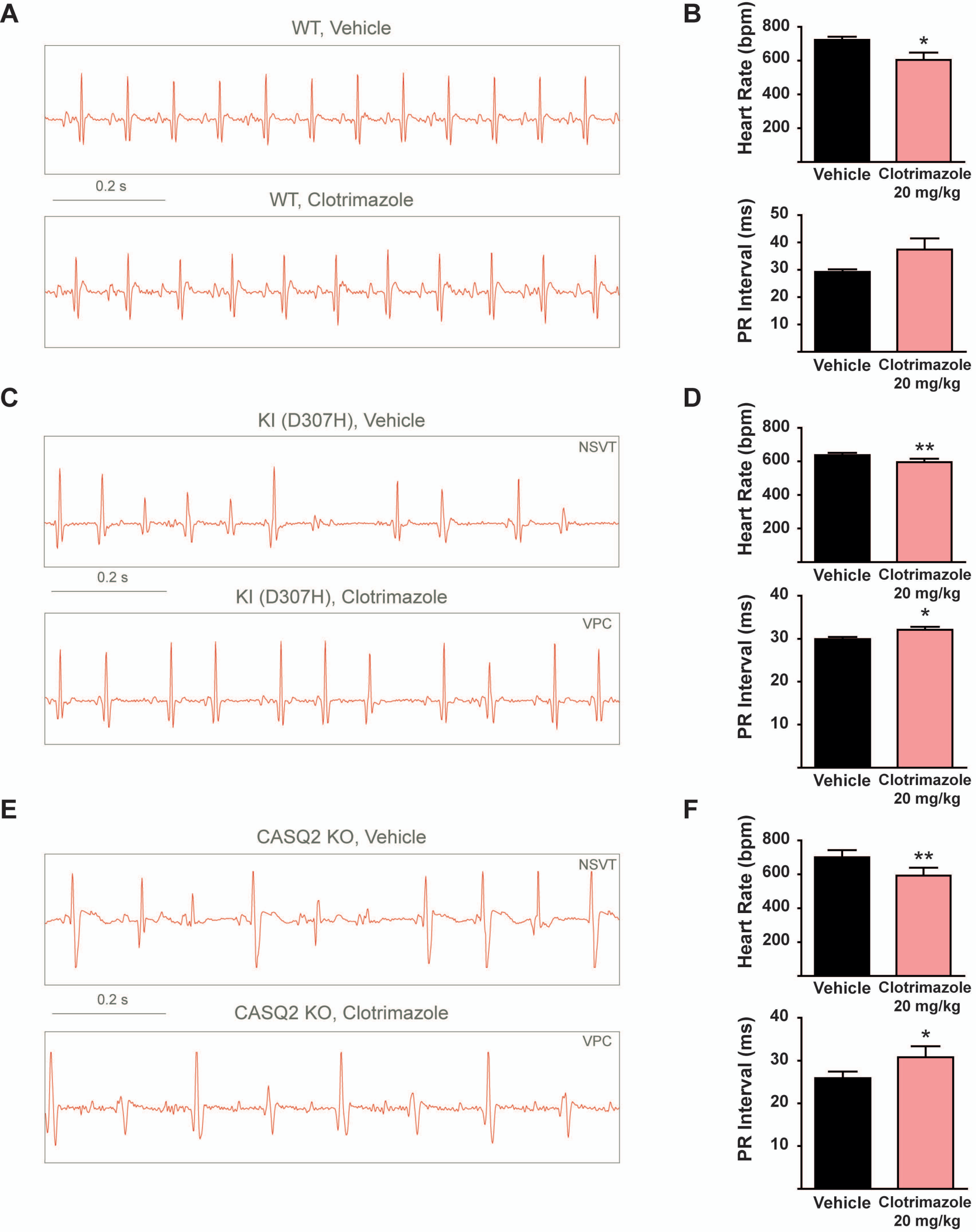
A
WT, Vehicle
0.2 s
WT, Clotrimazole
B
Heart Rate (bpm)
800
600
400
200
*
Vehicle
Clotrimazole 20 mg/kg
PR Interval (ms)
50
40
30
20
10
Vehicle
Clotrimazole 20 mg/kg
C
KI (D307H), Vehicle
NSVT
0.2 s
KI (D307H), Clotrimazole
VPC
D
Heart Rate (bpm)
800
600
400
200
**
Vehicle
Clotrimazole 20 mg/kg
PR Interval (ms)
40
30
20
10
*
Vehicle
Clotrimazole 20 mg/kg
E
CASQ2 KO, Vehicle
NSVT
0.2 s
CASQ2 KO, Clotrimazole
VPC
F
Heart Rate (bpm)
800
600
400
200
**
Vehicle
Clotrimazole 20 mg/kg
PR Interval (ms)
40
30
20
10
*
Vehicle
Clotrimazole 20 mg/kg

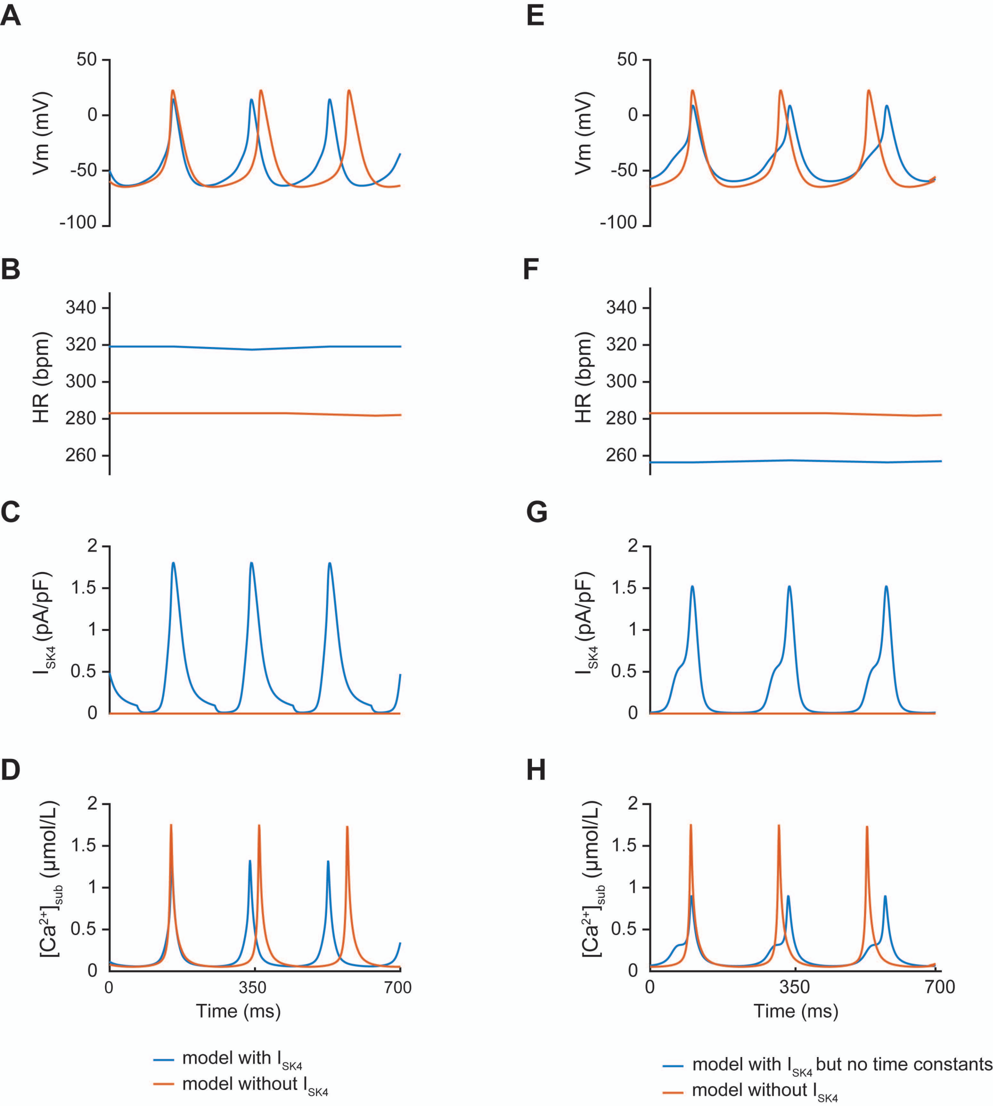

A
Vm (mV)
50
0
-50
-100
B
HR (bpm)
340
320
300
280
260
C
I_{SK4} (pA/pF)
2
1.5
1
0.5
0
D
$[Ca^{2+}]_{sub}$ (µmol/L)
2
1.5
1
0.5
0
0
350
700
Time (ms)
model with I_{SK4}
model without I_{SK4}
E
F
G
H
model with I_{SK4} but no time constants
model without I_{SK4}
